# Protein deubiquitylase USP3 stabilizes Aurora A to promote proliferation and metastasis of esophageal squamous cell carcinoma

**DOI:** 10.1186/s12885-021-08934-x

**Published:** 2021-11-10

**Authors:** Ke Shi, Jin Zhong Zhang, Liang Yang, Ning-Ning Li, Ying Yue, Xiu-Hong Du, Xiu-Zhi Zhang, Yu Cheng Lu, Dan Guo

**Affiliations:** 1 Department of Biochemistry and Molecular Biology, Henan Medical College, Zhengzhou, China; 2 Henan No.2 Provincial People’s Hospital, Henan Medical College Hospital Workers, Zhengzhou, China; 3 Central Laboratory, Linyi People’s Hospital, Linyi, Shandong China

**Keywords:** Aurora A, USP3, Ubiquitination, EMT, Esophageal squamous cell carcinoma

## Abstract

Aurora A kinase is a cell cycle regulator that is dysregulated in several different malignancies. Nevertheless, its regulatory mechanisms are still not fully understood. Here, we report that ubiquitin specific peptidase 3 (USP3) promotes proliferation and metastasis of esophageal squamous cell carcinoma (ESCC) cells by mediating deubiquitination of Aurora A. Analysis of human clinical samples indicated that USP3 and Aurora A are highly expressed in ESCC. Cellular experiments confirmed that high expression of USP3 and Aurora A in ESCC cells promoted malignant cell proliferation and invasion. In this mechanism, USP3 leads to suppression of Aurora A ubiquitination, resulting less proteasome degradation. We constructed the deubiquitinated mimetic K143R of Aurora A and found that K143R significantly promoted the proliferation and invasion of ESCC cells and was not regulated by the deubiquitination of USP3. Moreover, Aurora A K143R potentiated the kinase activity of Aurora A in ESCC cells. Thus, our findings demonstrate that the tumorigenic feature of ESCC is in part mediated by USP3-facilitated deubiquitination of Aurora A.

## Introduction

Esophageal squamous cell carcinoma (ESCC) an extremely common gastrointestinal malignancy that carries with its high morbidity and mortality. It is currently the 9th cause of highest global cancer incidence and the 6th cause of highest total mortality rate. In 1990, ESCC was the 7th largest cancer, climbing up the ranks to the 6th in 2013. An aging population along with a massive population growth are drivers of the increased occurrence of ESCC [8, 9]. Squamous cell carcinoma and adenocarcinoma are the main ESCC pathological subtypes. Patients are often diagnosed with advanced ESCC upon their first clinical presentation as the early signs of ESCC are often subtle, a situation compounded by a lack of effective means of early diagnosis. Tumor invasion and metastasis are the primary causes of death in patients with ESCC [21]. Invasion and metastasis are dependent on the activation of proto-oncogenes and the inactivation of tumor suppressor genes, as well as several other pathways involved in cell proliferation and apoptosis. The increasing research in the genetics and epigenetics of ESCC [3] offers promise of discovering new targets and new strategies for the diagnosis and treatment of this disease.

In recent years, many studies have shown that Aurora A, a serine/threonine kinase involved in mitosis and associated with centrosomes, is highly expressed in ESCC and promotes proliferation, invasion and metastasis of ESCC cells [19, 22]. A loss of cell-to-cell adhesion is the primary cause of tumor cell migration and metastasis [12]. Weakened cell adhesion promotes malignant cell detachment from the surrounding cells, which is the first step of tumor invasion and metastasis. However, the mechanism of how Aurora A affects ESCC cell adhesion has yet to be elucidated. Therefore, this study seeks to explore the effect of high Aurora A expression on adhesion of ESCC cells.

Ubiquitin-specific protease 3 (USP3) is a member of the USP family and is located on the human chromosome 15q22.3 and consists of 520 amino acids. USP3 contains a highly conserved cysteine residue and two highly conserved histidines. Residues are involved in cellular processes by mediating deubiquitination of target proteins [16]. USP3 is able to regulate monoubiquitinated H2A and H2B levels, and USP3 knockdown in U2OS cells leads to delay in the S-phase of the cell cycle as well as increased accumulation of ionizing radiation-induced DNA damage [11]. In Hela cells exposed to UV-induced DNA damage, USP3 functions to deubiquitinate H2A and γ-H2AX by targeting RNF168, thereby reducing the accumulation of BRCA1 at DNA breaks [15]. Overexpression of wild-type USP3 in HepG2 cells deubiquitinated MDM2 and up-regulated the protein level of MDM2. In normal hepatocyte cell lines HL-7702, overexpression of USP3 did not have the function of stabilizing MDM2, and CO-IP experiments demonstrated that there was no interaction between them, indicating that the deubiquitination of MDM2 by USP3 only occurred in hepatoma cells and promoted the proliferation of HepG2 cells.

Given the important role of multiple DUBs in cancer-associated signaling pathways, little is known regarding the roles of USP3 and Aurora A in tumor invasion and metastasis. The ubiquitination modification site and the regulatory effect of USP3 on centrosome and tumor function have yet to be reported. This study confirms that USP3 interacts with Aurora A in order to inhibit ubiquitination of the Aurora A K143 locus. Both USP3 and Aurora A can promote the proliferation, invasion and metastasis of ESCC.

## Method and materials

### Cell lines and materials

The ECa109 cell line was purchased from ATCC and cultured in DMEM (Gibco, USA) supplemented with 10% FBS (Gibco, USA). Antibodies against Aurora A (Proteintech, USA), USP3 (Proteintech, USA), GAPDH (Cell Signaling Technology, USA), Flag (Sigma, USA), HA (Abcam, USA) were used. Propidium iodide (PI) reagent was purchased from Sigma, USA.

### Immunoprecipitation

The ECa109 cells expressing Flag-USP3 or HA-Aurora A were first washed on a 6 cm culture plate with PBS buffer. The cells on each plate were then scraped off and were lysed with 1 ml of ice-cold RIPA lysis buffer. Cell lysates were collected and anti-Flag beads (Sigma, USA) or anti-HA beads (Sigma, USA) were added. The resulting immunoprecipitate was shaken overnight at 4 °C. The beads were washed and loaded into the loading buffer, and the levels of bound proteins were detected by SDS-PAGE followed by western blotting.

### RNA interference

Aurora A and USP3 gene knockdown were performed by siRNA in this study. The siRNA sequences used are as follows: Aurora A–siRNA (5′- CACAUACCAAGAGACCUACAA-3′, 5′-UUGUAGGUCUCUUGGUAUGUG-3′), USP3–siRNA (5′-CCAACCAUAAGAAAUCAGAAA-3′, 5′- UUUCUGAUUUCUUAUGGUUGG-3′), Negative control-siRNA (5′- UUCUCCGAACGUGUCACGUTT-3′, 5′-ACGUGACACGUUCGGAGAATT-3′).

### RT-PCR

TRIzol reagent was used to isolate total RNA from frozen tissue samples and cultured cells. RNA was reverse transcribed into cDNA using SYBR Premix Ex Taq (Takara, USA). Primers used included: Aurora A (Forward-GGATATCTCAGTGGCGGACG, Reverse-GCAATGGAGTGAGACCCTCT), USP3 (Forward-GCTTTCTTTGACGCAAGGGC, Reverse-GGACCGGCACACGCTG). The RT-PCR primers for the EMT pathway markers and AP-2α [25], NF-κB [2], AKT-RAS [23] downstream genes utilized previously published sequences.

### CCK-8 analysis

Each group of treated ECa109 cells was collected, seeded onto 96-well plates at a density of 10^4^ cells per well, and cultured for 24–96 h. 10 μL CCK-8 solution (Beyotime Inst Biotech, China) was added to each well at 24, 48, 72 and 96 h, and then cell viability was measured by using a 450 nm absorbance microplate reader.

### Colony formation analysis

The treated ECa109 cells were seeded into a 12-well plate of 200 cells per well, and then cultured at 37 °C for about 14 days. The cells on the plate were washed twice with PBS solution and fixed with paraformaldehyde for 20 min. 0.1% crystal violet was then used to stain the cells for 15 min. Cell colonies were finally counted and statistically analyzed.

### Cell cycle analysis

ECa109 cells cultured in 12-well plates were collected and fixed in 70% ethanol overnight at − 20 °C. It was then resuspended in PBS and incubated with 10 mg/mL RNase and 1 mg/mL propidium iodide for 15 min at 37 °C. DNA content analysis was performed by flow cytometry (BD Biosciences, USA). Modfit software was used to analyze cell distribution at different cell cycle stages.

### Wound healing analysis

Equal amounts of differently treated ECa109 cells were seeded in six well plates until confluence was achieved. Linear wounds were carefully made with 200 μL pipette tips. Cell debris was removed by washing with PBS, and cells were further incubated. Photos of the wound monolayer were then taken at 0- and 24-h post-injury.

### Cell invasion detection

To measure the invasion of treated ECa109 cells, Transwell (Corning Incorporated, USA) chambers was placed in the wells of a 24-well culture plate. In the lower chamber, 600 μL of DMEM medium containing 10% fetal bovine serum was added. Serum-free DMEM and treated cells were added to the upper chamber. After 24 h of culture, the migrated cells were fixed with paraformaldehyde and stained with 0.1% crystal violet. The number of cells were quantified by counting three randomly selected independent fields under the light microstructure.

### Statistical analysis

All statistical analyses were performed using SPSS 19.0. All experimental data are expressed as the mean ± SD of at least three independent experiments. The One-way ANOVA test was used to analyze differences between experimental groups. All differences were considered statistically significant at *P* < 0.05.

## Results

### High expression of Aurora A and USP3 in human ESCC

**Fig. 1:**
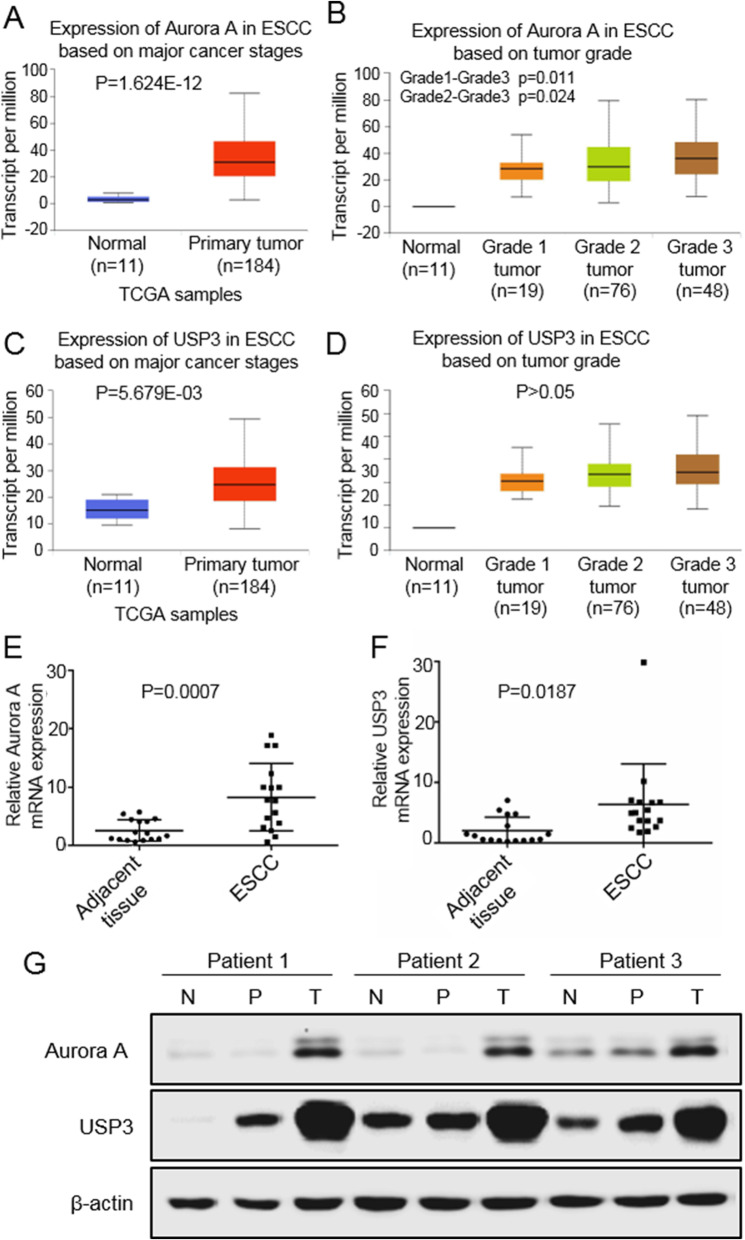
The TCGA database and ESCC specimens validate the expression of USP3 and Aurora A. **A** Aurora A is highly expressed in ESCCrelative to normal tissues in the TCGA database. **B** Aurora A is highly expressed in high grade ESCC. **C** USP3 is highly expressed in ESCCrelative to normal tissues. **D** USP3 was not significantly expressed in high and low graded ESCC. **E** Expression of Aurora A mRNA in 16 human ESCC specimens. **F** Expression of USP3 mRNA in 16 human ESCC specimens. **G** Aurora A and USP3 protein expression in 3 typical ESCC specimens

We first studied human ESCC samples. Results based on the TCGA database showed that Aurora A was highly expressed in human ESCC tissues (Fig. 1A), and that high expression of Aurora A correlated to a high ESCC grade (Fig. 1B). On the other hand, USP3 was highly expressed in ESCC (Fig. 1C) but did not have a significant correlation with tumor grade (Fig. 1D). We collected 16 cases of ESCCand its corresponding adjacent tissues for mRNA detection. RT-PCR results showed that mRNA levels of Aurora A and USP3 were highly expressed in ESCC compared to adjacent tissues (Fig. 1E and F). The tissue samples were then lysed and protein content extracted in order to determine the protein levels of Aurora A and USP3. We found that Aurora A and USP3 proteins were highly expressed in ESCC tissues (Fig. 1G). In conclusion, these findings indicate that Aurora A and USP3 are highly expressed in human ESCCtissues and are associated with poor tumor grade.

### Increased proliferation of ESCC cell lines by Aurora A and USP3

**Fig. 2:**
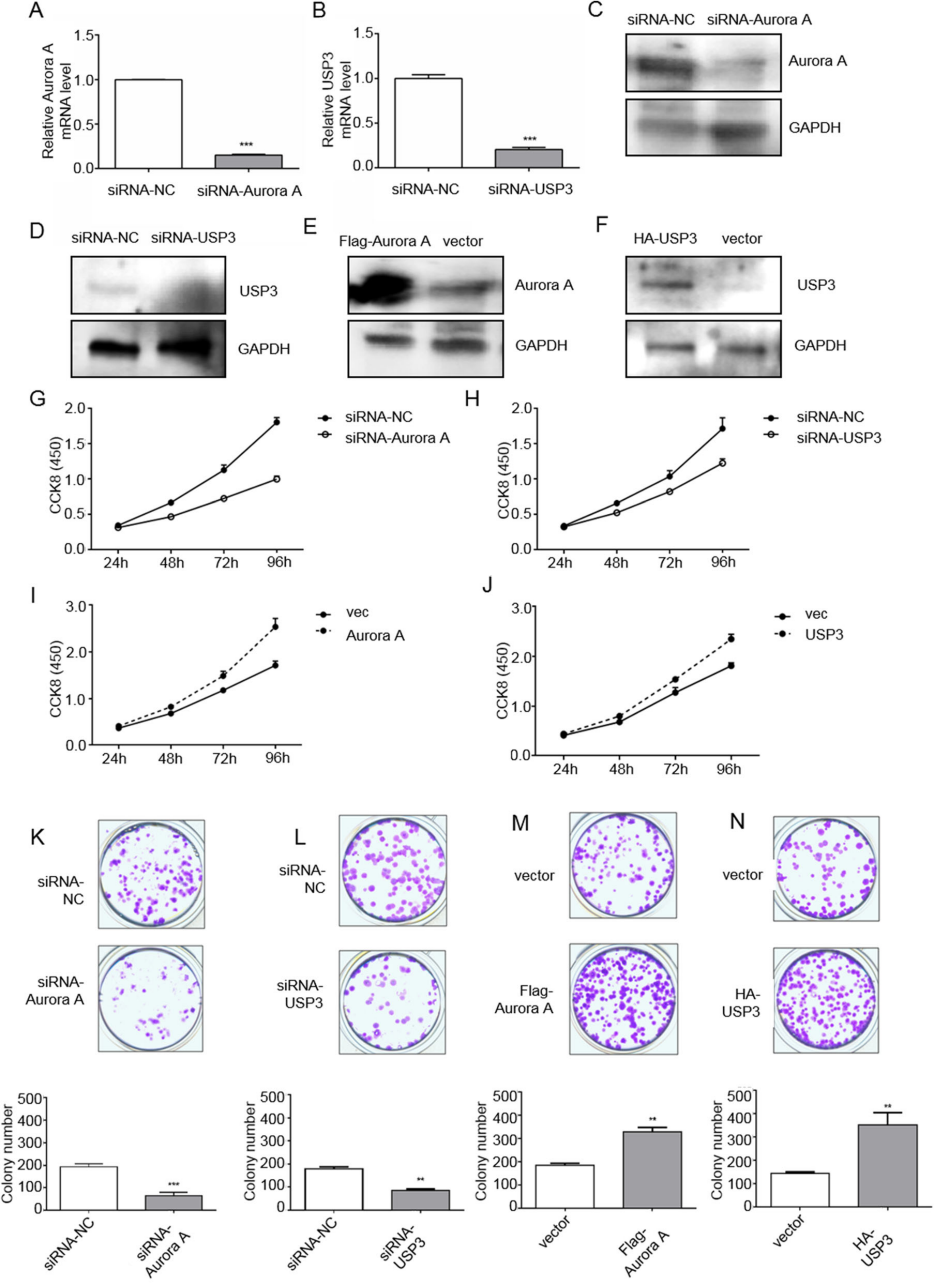
USP3 and Aurora A promote proliferation of ESCCcells. **A**-**B** Aurora A and USP3 were knocked down by siRNA in ESCCcell line ECa109 cells, respectively, and knockdown efficiency was measured by qPCR. **C**-**D** Aurora A and USP3 were knocked down by siRNA in ECa109 cells, and the protein was knocked out in the cells by western blot. **E**-**F** Overexpression of Aurora A and USP3 in ECa109 cells, and Western blot analysis confirmed that proteins were highly expressed in cells. **G**-**J** CCK8 confirmed that Aurora A and USP3 promoted the proliferation of ECa109 cells. **K**-**L** After Aurora A and USP3 knockdown by siRNA in ECa109 cells, cells were plated in 12-well plates, cultured for 14 days, and then stained with crystal violet. **M**-**N** After over-expressing Flag-Aurora A and HA-USP3 in ECa109 cells, cells were plated in 12-well plates, cultured for 14 days, and then stained with crystal violet

Next, we investigated the function of Aurora A and USP3 in ESCC cell lines. We selected ESCC cell line ECa109 as a research model and performed siRNA knockdown and overexpression of Aurora A and USP3 in the ECa109 cells. Knockdown and overexpression of Aurora A (Fig. 2A, C and E) and USP3 (Fig. 2B, D and F) in ECa109 cells were detected at mRNA and protein levels. In ECa109 cells, knockdown of Aurora A and USP3 using siRNA significantly slowed the proliferation of ECa109 cells (Fig. 2G and H). Overexpression of Aurora A and USP3 increased the rate ECa109 cell proliferation (Fig. 2I and J). Similarly, colony formation assays confirmed a decrease in the number of clones in Aurora A and USP3 knockdown ECa109 cells (Fig. 2K and L), and an increase in the number of Aurora A and USP3 overexpressed ECa109 cell clones (Fig. 2M and N). Our data confirms that Aurora A and USP3 were able to promote proliferation of ESCC cells.

### Aurora A and USP3 promote invasion and metastasis of ESCC cells

**Fig. 3:**
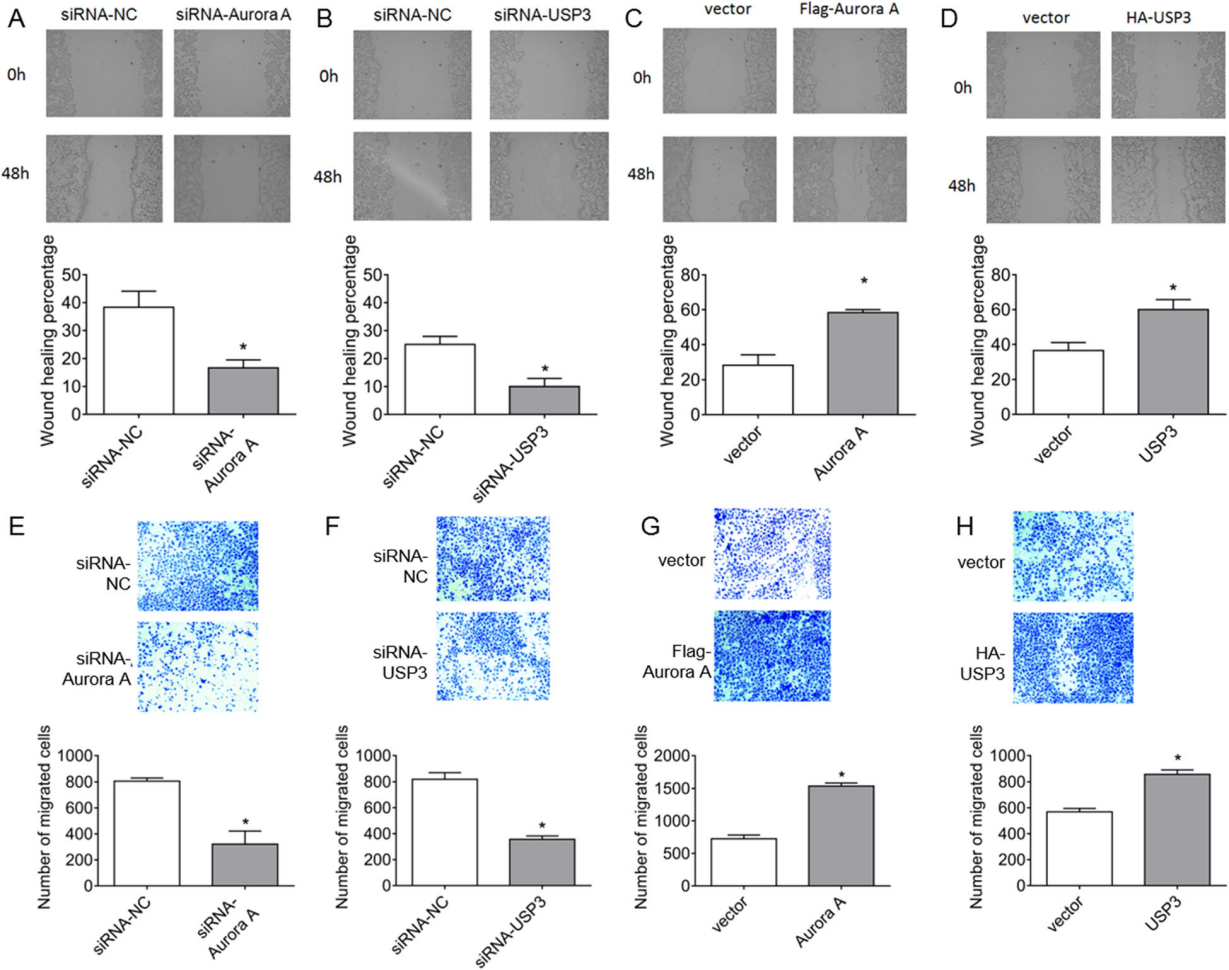
USP3 and Aurora A promote invasion and metastasis of ESCC cells. **A**-**B** ECa109 cells were plated in 6-well plates. After siRNA knocked out Aurora A and USP3, wound healing experiments were performed to observe the degree of scratch healing. **C**-**D** After overexpression of Flag-Aurora A and HA-USP3, wound healing experiments were performed to observe the degree of healing of the scratches. **E**-**F** ECa109 cells were plated in 6-well plates, and after knocking down Aurora A and USP3 with siRNA, transwell experiments were performed and the number of cells passing through transwell was counted. **G**-**H** After overexpression of Flag-Aurora A and HA-USP3, transwell experiments were performed and the number of cells passing through transwell was counted

We then studied the role of Aurora A and USP3 in the invasion and metastasis of ESCC cells. ECa109 cells were plated in 6-well plates. The wound healing assays examined the influences of Aurora A and USP3 knockdown on migration ability. The migration distances of Aurora A (Fig. 3A) and USP3 (Fig. 3B) siRNA-treated ECa109 cells were significantly reduced compared to the control siNC cells. Conversely, ECa109 cells that overexpressed Aurora A (Fig. 3C) and USP3 (Fig. 3D) were found to have significantly increased migration distances. In the invasion and metastasis assays, we tested the invasion and migration ability of treated ECa109 cells through Transwell assays. The numbers of invading cells in Aurora A (Fig. 3E) and USP3 (Fig. 3F) siRNA-treated ECa109 cells were significantly reduced compared to the control siNC cells. After overexpression of Aurora A (Fig. 3G) and USP3 (Fig. 3H), the numbers of invading ECa109 cells increased significantly. Our data indicates that Aurora A and USP3 upregulation enhances the ability of ESCC cells to invade and metastasize.

### USP3 improves Aurora A protein stability

**Fig. 4:**
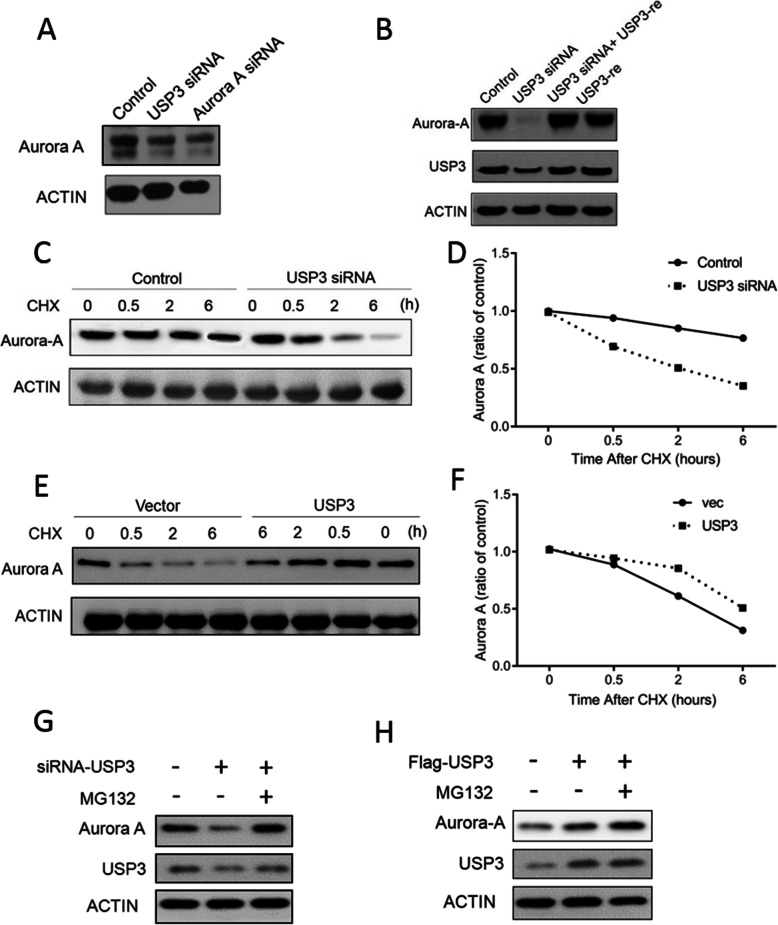
USP3 regulates Aurora A protein stability, thereby modulating Aurora A protein levels. **A** Aurora A levels were measured after ECa109 cells were knocked down of USP3. **B** USP3 was overexpressed while knocking down USP3, and Aurora A levels were detected. **C**-**D** After knocking down USP3, the protein degradation promoter CHX was added to detect Aurora A levels. **E**-**F**. After overexpressing USP3, the protein degradation promoter CHX was added to detect Aurora A levels. **G**-**H**. After knocking down and overexpressing USP3, the protein degradation inhibitor MG132 was added to detect Aurora A levels

USP3 in ESCC cells has a regulatory effect on Aurora A. After siRNA knockdown of USP3 in ECa109 cells, Aurora A protein levels were decreased (Fig. 4A), and after overexpression of USP3, Aurora A protein levels were restored to normal levels (Fig. 4B). ECa109 cells that had USP3 knocked-down treated with cycloheximide (CHX) were noted to have significantly decreased Aurora A protein stability (Fig. 4C and D). After overexpression of USP3, the protein stability of Aurora A in CHX-treated ESCC cells was significantly increased (Fig. 4E and F). Treatment of ECa109 cells with the proteasome inhibitor MG132 reversed the inhibitory effect of USP3-siRNA on Aurora A protein (Fig. 4G). After overexpressing USP3, MG132 no longer increased Aurora A protein levels (Fig. 4H). Therefore, USP3 enhances the stability of Aurora A protein by inhibiting the proteasome degradation pathway of Aurora A protein.

### USP3 interacts with Aurora A and reduces ubiquitination of Aurora A

**Fig. 5:**
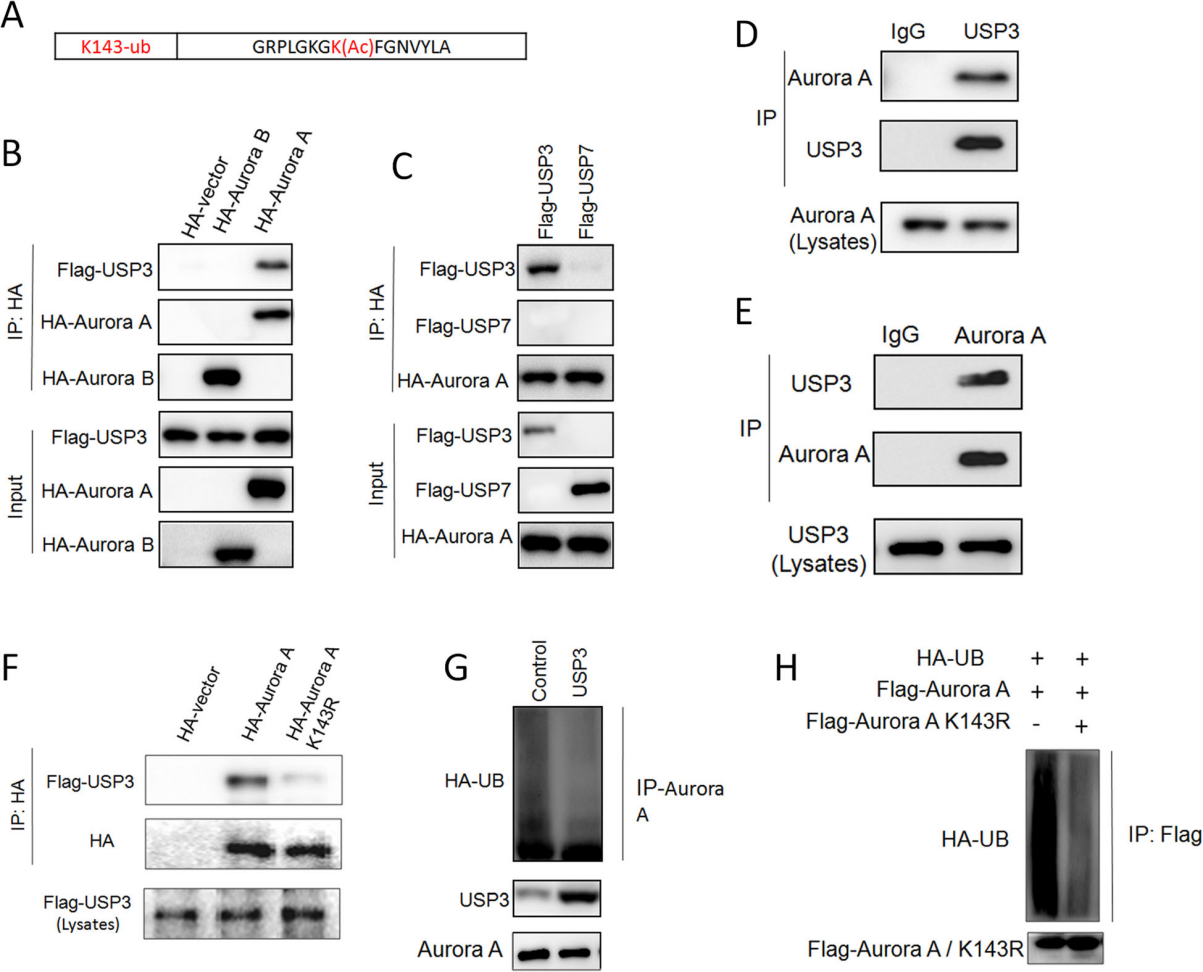
USP3 interacts with Aurora-A to inhibit ubiquitination of Aurora A K143. **A** Aurora A ubiquitinated mutant Aurora A K143R was constructed and expressed in ECa109 cells. **B**-**C** Exogenous overexpression of Flag-USP3 and HA-Aurora A, co-IP experiments demonstrated the interaction. **D**-**E** Endogenous co-IP demonstrates the interaction between USP3 and Aurora A. **F** After the mutation Aurora A K143 was to K143R, the interaction between USP3 and Aurora A was reduced. **G** The ubiquitination of Aurora A was detected after overexpression of USP3. **H** Compare the ubiquitination levels of Aurora A K143R and Aurora A

Proteasome degradation of Aurora A is dependent on the ubiquitination of Aurora A. The ubiquitination site of Aurora A was predicted to be at the lysine molecule at position 143, using the Uniprot database (Fig. 5A). We found that there was a protein interaction between exogenously expressed USP3 and Aurora A, not Aurora B (Fig. 5B). Similarly, immunoprecipitation of Aurora A protein showed that Aurora A binds USP3 instead of USP7 (Fig. 5C). Endogenous co-immunoprecipitation assays showed an interaction between USP3 and Aurora A (Fig. 5D and E). We mutated the predicted Aurora A lysine ubiquitination site to arginine (K143R), and the co-immunoprecipitation experiment demonstrated a decrease in the interaction between Aurora A K143R and USP3 (Fig. 5F). In vitro ubiquitination experiments showed that overexpressing USP3 significantly inhibited Aurora A ubiquitin binding (Fig. 5G). The level of ubiquitination of Aurora A K143R was significantly lower than that of Aurora A (Fig. 5H). Our data confirm that USP3 interacts with Aurora A and mediates deubiquitination of the Aurora A K143 locus.

### USP3 promotes proliferation and metastasis of ESCC cells by modulating Aurora A K143 ubiquitination

**Fig. 6:**
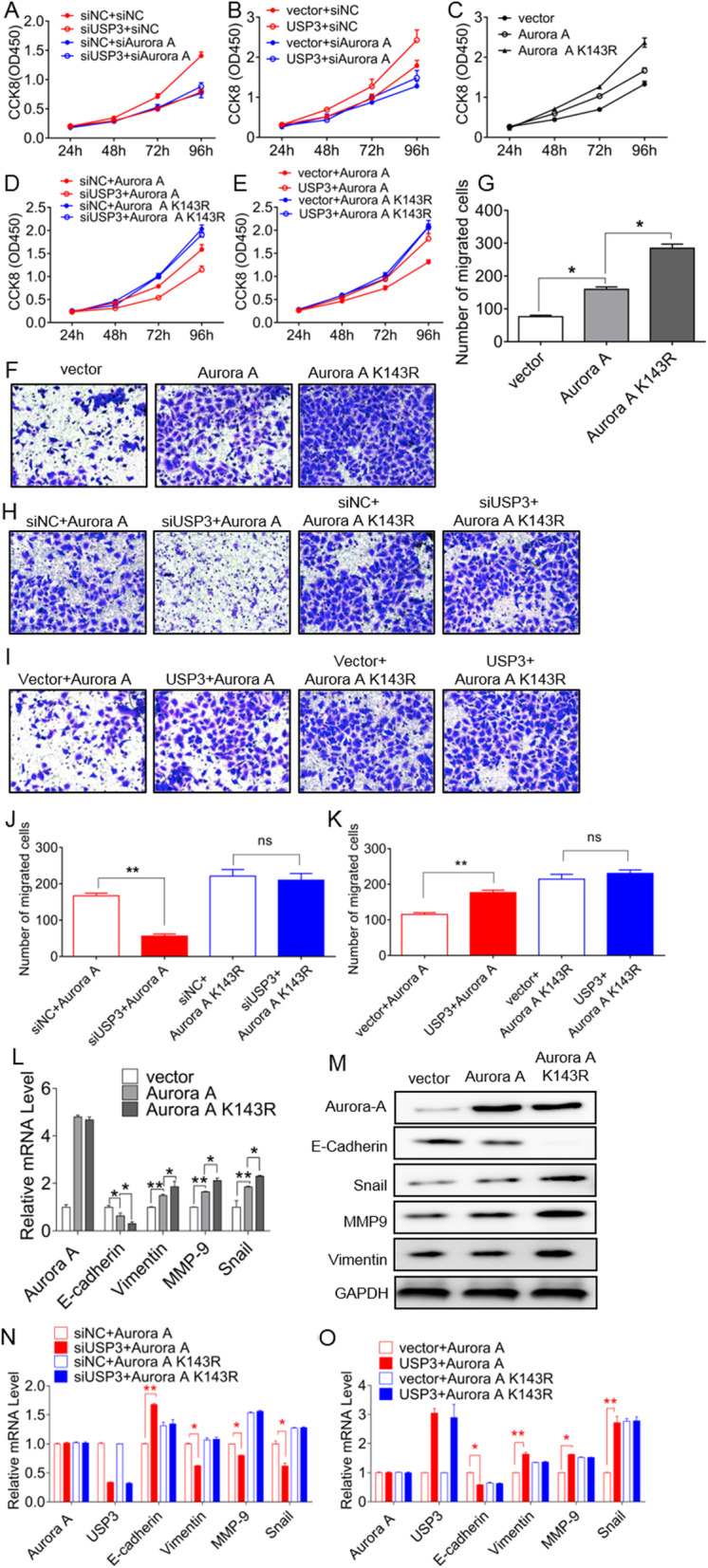
USP3 promotes proliferation and metastasis of ESCC cells and promotes EMT through Aurora A K143. **A**-**B** After knocking down Aurora A with siRNA, USP3 attenuated changed in cell proliferation. **C** When ECa109 cells overexpressed Aurora A K143R, the cell proliferation rate was accelerated. **D**-**E** After overexpression of the Aurora A K143R mutant in ECa109 cells, USP3 reduced the effect on cell proliferation. **F**-**G** After overexpression of Aurora A K143R by ECa109 cells, cell invasion and metastasis increased. **H**-**K** After overexpression of the Aurora A K143R mutant, USP3 had a reduced effect on cell invasion and metastasis. **L**-**M**. mRNA and protein assays demonstrated that Aurora A and Aurora A K143R promoted the EMT process. **N**-**O**. After overexpression of the Aurora A K143R mutant, USP3 attenuated changes in the EMT process

The proliferation of ESCC by USP3 is dependent on Aurora A K143 ubiquitination. Knockdown (Fig. 6A) and overexpression (Fig. 6B) of USP3 respectively inhibited and promoted the proliferation of ESCC, and these phenomena were no longer significant after Aurora A knockdown. Aurora A deubiquitinated mimetic Aurora A K143R promotes ESCC proliferation (Fig. 6C). After knockdown of USP3 in ECa109 cells, overexpression of Aurora A K143R reversed the inhibitory effect of USP3-siRNA on ESCC cells (Fig. 6D). Similarly, in ECa109 cells overexpressing both Aurora A K143R and USP3 experienced attenuation in degree of cell proliferation (Fig. 6E). On the other hand, overexpression of Aurora A K143R significantly promoted invasion and metastasis (Fig. 6F and G) of ECa109 cells. After overexpression of Aurora A K143R, USP3-siRNA inhibited cell invasion (Fig. 6H and J) and USP3 significantly attenuated cell invasion (Fig. 6I and K). We examined mRNA and protein expression of EMT-related markers and found that overexpression of Aurora A K143R significantly promoted the process of EMT (Fig. 6L and M). Similarly, the promotion of EMT by ECP109 cells by USP3 was also dependent on the Aurora A K143 site (Fig. 6N and O). Therefore, USP3 promotes the proliferation and metastasis of ESCC by regulating Aurora A K143 ubiquitination.

### USP3-dependent Aurora A deubiquitination stabilizes Aurora A

**Fig. 7:**
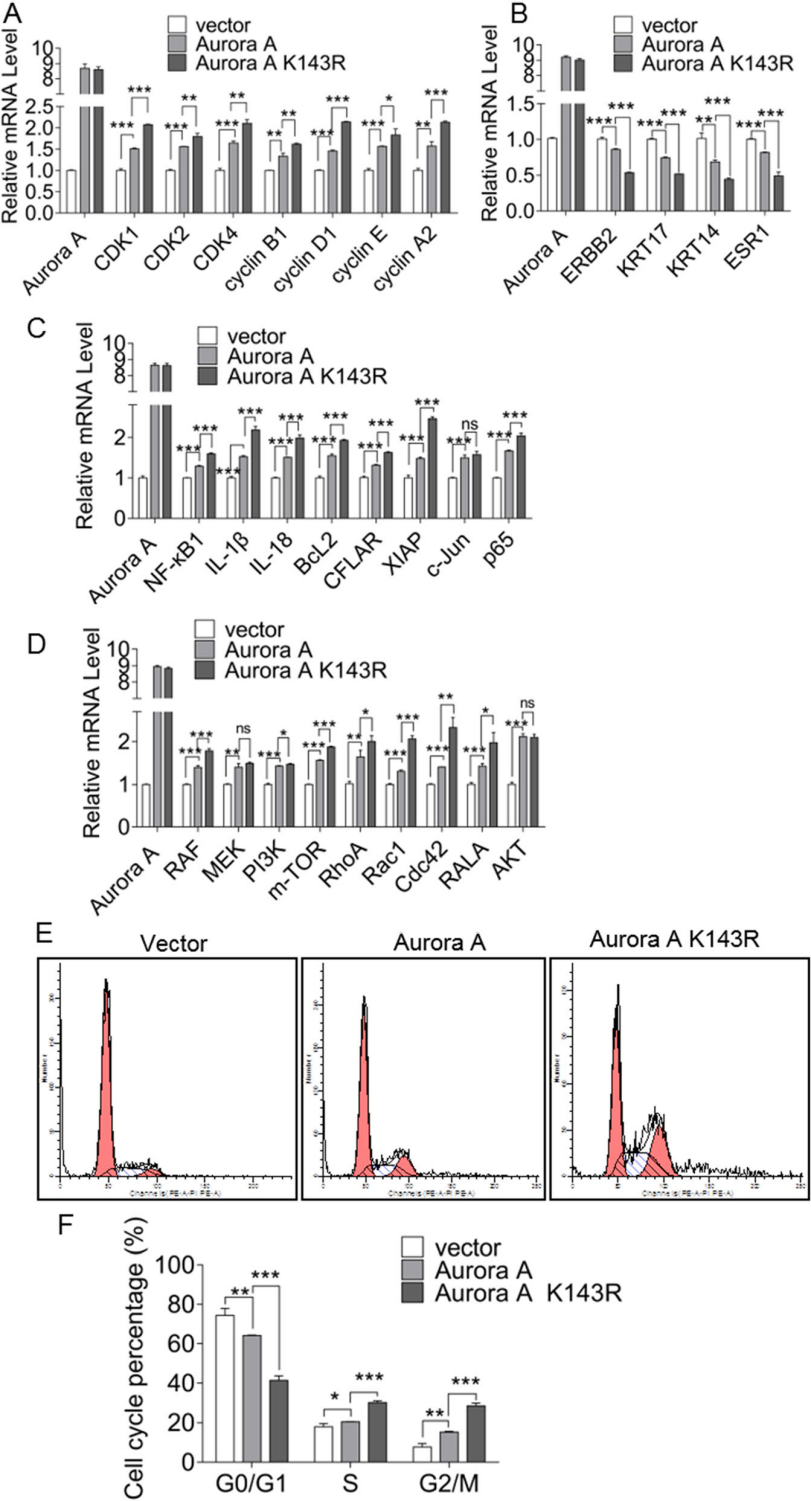
The activity of the ubiquitinated mutant Aurora A K143R is enhanced. **A** The mRNA expression of cell cycle-related downstream genes in ECa109 cells overexpressed Aurora A and Aurora A K143R was detected. **B** The mRNA expression of AP-2α-related downstream genes under Aurora A and Aurora A K143R overexpression was compared. **C** IκBα-NF-κB-related downstream genes mRNA expression was detected. **D** Overexpression of Aurora A and Aurora A K143R was performed to detect the expression of AKT-RAS-related downstream gene mRNA. **E**-**F** Compare the effects of Aurora A and Aurora A K143R on cell cycle changes

The Aurora A kinase activity of the deubiquitinated mimetic K143R was significantly enhanced. After overexpression of Aurora A K143R in ECa109 cells, mRNA expressions of Aurora A-dependent cell cycle progression-associated genes were significantly increased (Fig. 7A), and Aurora A K143R significantly promoted ESCC cell cycle progression (Fig. 7E and F). At the same time, Aurora A K143R down-regulated the activity of AP-2α tumor suppressor and exhibited a tumor-promoting phenotype (Fig. 7B). Since Aurora A phosphorylates IκBα to activate the transcription factor NF-κB, Aurora A K143R resulted in an increase in mRNA levels of NF-κB downstream related genes (Fig. 7C). In addition, Aurora A K143R also activated the AKT-RAS signaling pathway (Fig. 7D). Thus, USP3-dependent deubiquitination of Aurora A in ESCC cells enhances the kinase activity of Aurora A.

## Discussion

ESCCis one of the most common malignant tumors of the digestive tract. Its morbidity and mortality have been steadily increasing over the recent years. The mortality rate of ESCCwas ranked 6th in the overall global cancer mortality rate, with the main cause of death in ESCC being tumor metastasis and recurrence [17]. Therefore, studying the molecular mechanisms involved in the metastasis of ESCCwill potentially serve to uncover a target for its treatment and ultimately improve the survival rate of cancer patients.

Tumor metastasis is a complex multi-step process that involves three major steps: adhesion, matrix dissolution and migration [1, 14]. The ability of cells to adhere to cells plays an important role in tumor invasion and metastasis. Studies have found that changes in certain cell adhesion molecules on the surface of tumor cells enhance the adhesion of cells to the outer matrix. When adhesion between cells is weakened, there is a tendency for cancer cells to detach and migrate away from the basement membrane [7, 18]. This process serves as the initial steps for tumor invasion and metastasis.

As a member of the Aurora kinase family, Aurora-A plays an important regulatory role in the maturation of cell centrosomes, the establishment of bipolar spindles and the normal isolation of chromosomes [22]. Previous studies have found that Aurora-A is highly expressed in ESCC, and can promote the proliferation, angiogenesis, invasion and metastasis of ESCC cells, and participate in the regulation of the expression of a variety of related protein molecules [24]. Pezzani et al. showed that after treatment with Aurora kinase inhibitor VX-680, the expression of Aurora A decreased and the expression of E-cadherin increased with the increase of VX-680 concentration [13]. The above studies suggest that high expression of Aurora-A may affect the adhesion of tumor cells to cells by affecting E-cadherin. This is consistent with our findings.

Ubiquitin specific protease 3 (USP3) is a significant deubiquitinating enzyme and is a member of the USPs family, a specific group of proteases that cleave an ubiquitin chain linked by a proline residue [6]. USP3 also inhibits the type I interferon pathway in antiviral immune responses by deubiquitinating the RIG-I receptor [4]. In the USP3 knockout mouse model, monoubiquitinated H2A and H2B and spontaneous chromosomal breaks in tissue cells were significantly increased, while in vivo hematopoietic stem cell reserves and mouse lifespan were significantly reduced [11].

It indicates that USP3 plays an important role in maintaining the physiological state of the body and the homeostasis under stress response [10]. In our study, we found that USP3 interacted with Aurora A to reduce the ubiquitination level of Aurora A and inhibited the proteasome degradation pathway of Aurora A. We constructed the deubiquitinated mimetic K143R of Aurora A and found that K143R significantly promoted the proliferation and invasion of ESCC cells, and was not regulated by the deubiquitination of USP3. Moreover, Aurora A K143R potentiated the kinase activity of Aurora A in ESCC cells. Thus, our findings demonstrated that USP3 deubiquitinated Aurora-A induced tumorigenic features of ESCC.

Current research indicates that the USP3 protein can participate in various life activities in cells, such as cell proliferation, cell cycle and DNA damage repair, which are closely related to the occurrence and development of diseases [5, 20]. The molecular mechanisms underlying the development and progression of USP3 disease are still unclear. Therefore, the molecular function of USP3 in cells and its relationship with disease requires further clarification, potentially uncovering a molecular target that may aid diagnosis and treatment of clinical diseases.

## Data Availability

All data generated or analyzed during this study are included in this article.
